# Intestinal Anastomosis with Segmented Rubber Rings

**Published:** 1889-12

**Authors:** 


### INTESTINAL ANASTOMOSIS WITH SEGMENTED RUBBER RINGS.

At the recent meeting of the Southern Surgical and Gynecological Association, at Nashville, Dr. A. V. L. Brokaw, of St. Louis, Mo., read a paper entitled "Intestinal Anastomosic Operations with Segmented Rubber Rings, with some Practical Suggestions as to their Use in other Surgical Procedures." The paper considered in detail the results obtained in an experimental study of all the anastomotic operations, and an original technique and application of segmented rubber rings in such operations as gastrotomy, duodeno-cholecystotomy, jejuno-cholecystotomy, ileo-colostomy, and circular-enterorrhaphy. Reference at length was made to the author's success in closing very large wounds of the intestines by the use of a single segmented rubber ring, formed of eight sections of tubing. The ring, being introduced into the intestines, is bent evenly upon itself, and the apposition threads being tied, perfect, safe closure of the very largest wound is accomplished without stenosis following. The ring devised by the author, is very simply constructed by passing a doubled strand of catgut continuously through from four to eight short sections of rubber drainage-tubing, of a diameter from one-sixteenth to one-fourth of an inch. To the catgut within the rubber sections are tied the apposition threads. The segmented rubber rings are applied in the anastomotic operations in the same manner as Senn proposed, in the use of his bone plates. The advantage of segmented rubber rings over other procedures and devices, is the rapidity with which they may be made, during an operation if need be. In the absence of proper tubing, pieces of catheter could be substituted. For the closure of small wounds in the intestines, a new suture was proposed. Short rods of decalcified bone, one-sixteenth of an inch in thickness, and one-fourth of an inch in width, are perforated at points less than half an inch apart for the passage of the apposition threads, which are attached in this manner: a strand of chromacized catgut, or well-prepared juniper catgut, is doubled and a single knot made in the middle of this doubled cat-gut strand (silk may be used if preferred), the loop and end threads are passed through the small openings made in the decalcified bone-rods. Each thread and loop is threaded to separate needles, the rods introduced in the wound in the bowel, the needles passed from within outward less than a quarter of an inch from the wound margins, and the loops and single threads tied in pairs. For this method was claimed accurate, rapid, and safe closure of small wounds of the intestines. The author preferred a segmented rubber ring in the closure of large wounds.

A description of two new wholly absorbable apposition rings was given, which had experimentally shown excellent results. One was formed by decalcifying the long bones of chickens and young animals, the process of decalcifying being the same as for making bone drainage-tubes. With short sections of bone so prepared, and a double strand of catgut, the rings were made in a manner similar to the described segmented rubber rings. The second wholly absorbable ring was made of short sections of the arteries of large animals. After dissecting the arteries up from their sheaths, they are cut in short segments, boiled five minutes, then into the lumen of each section is pushed a glass rod, and they are then immersed in alcohol for a few days. When the rods are withdrawn, the hardened artery tubes are ready for use. With four to eight short sections of arteries so prepared, approximation rings are easily made by passing a double catgut strand continuously through the lumen, as described previously. These rings serve their purpose admirably, are very easily made, give a good-sized aperture, and are entirely absorbed in a few days. Experiments were made upon over fifty dogs by the above methods.

